# ZAK kinase at the crossroads of cellular stress: functional plasticity, human pathobiology, and precision therapeutics

**DOI:** 10.3389/fcell.2026.1909993

**Published:** 2026-09-10

**Authors:** Yeteng Xiong, Fei Luo, Yuhan Huang, Bingnan Li, Guanchuan Lin

**Affiliations:** 1 The First School of Clinical Medicine, Southern Medical University, Guangzhou, China; 2 School of Public Health, Southern Medical University, Guangzhou, China; 3 Department of Biochemistry and Molecular Biology, School of Basic Medical Sciences, and Guangdong Provincial Key Laboratory of Single Cell and Extracellular Vesicles, Southern Medical University, Guangzhou, China

**Keywords:** cellular pathology, isoform specificity, precision therapy, ribotoxic stress response, signaling, ZAK kinase

## Abstract

Mitogen-activated protein kinase kinase 20 (MAP3K20/ZAK) has emerged as a critical biophysical sensor, uniquely poised at the intersection of environmental stress and cellular fate. Its versatile signaling is primarily orchestrated by two structurally distinct splice variants: ZAKα, which drives the ribotoxic stress response (RSR) upon detecting ribosome collisions, and ZAKβ, which mediates homeostatic mechanotransduction. However, emerging evidence indicates that the biological roles of ZAK cannot be strictly categorized as either pathogenic or protective. It exhibits profound functional plasticity and engages non-canonical metabolic networks in a highly context-dependent manner. Consequently, dysregulated ZAK signaling acts as a powerful disease amplifier across diverse human pathologies, including aggressive malignancies, cardiac remodeling, systemic metabolic deterioration, and inherited myopathies. While targeting ZAK presents a compelling therapeutic opportunity, current broad-spectrum kinase inhibitors are heavily confounded by “on-target, off-tissue” toxicities, particularly within the epidermal barrier. In this review, we synthesize the structural mechanisms, downstream cascades, and complex pathobiology of ZAK. Furthermore, we discuss an emerging Frontier in ZAK pharmacology: the conceptual development of structure-guided, isoform-selective allosteric interventions designed to safely decouple pathogenic ZAKα signaling from essential ZAKβ-mediated tissue homeostasis.

## 1 Introduction

Mitogen-activated protein kinase kinase 20 (MAP3K20), commonly referred to as ZAK, represents a distinctive member of the mixed-lineage kinase (MLK) subgroup (Vind et al., 2020a; Guo et al., 2020; Ma et al., 2023). In the literature, this kinase has also been described as MLTK (Mixed Lineage Kinase-like Mitogen-Activated Protein Triple Kinase) or MRK ((Rao et al., 2019; Choi et al., 2005). While broad downstream target promiscuity is common among multiple MAP3Ks (Peterson et al., 2022), ZAK uniquely functions as a direct biophysical sensor. By converting physical cellular stresses (such as ribosome structural collisions or cytoskeleton mechanical strain) into a robust MAPK signal amplification effect, ZAK effectively bypasses the conventional requirement for complex receptor-driven signaling cascades (Wu et al., 2020; Rey et al., 2016).

**FIGURE 1:**
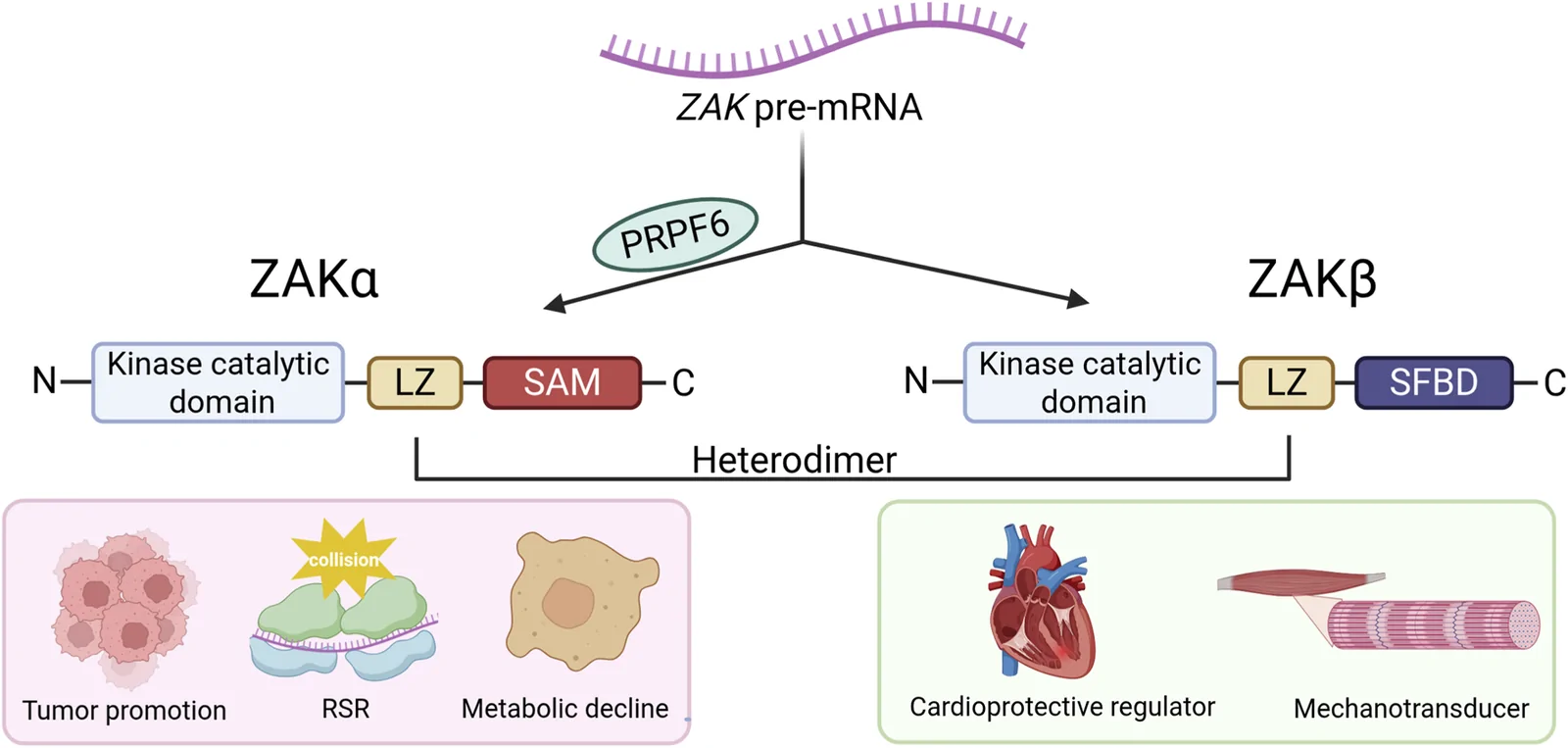
Structural dichotomy and context-dependent signaling of ZAK isoforms. The alternative splicing of ZAK yields two principal variants with distinct C-terminal structures: a SAM domain in ZAKα and an SFBD in ZAKβ. While ZAKα is classically associated with stress-amplifying cascades (e.g., RSR, metabolic decline, and tumor promotion) and ZAKβ canonically transduces homeostatic signals (e.g., mechanotransduction and cardioprotection), their biological outputs are not strictly binary. Importantly, the ultimate cellular fate driven by either isoform is highly plastic and governed by specific tissue microenvironments, rather than intrinsic ‘pathogenic’ or ‘protective’ paradigms. LZ, leucine-zipper; PRPF6, pre-mRNA processing factor 6; RSR, ribotoxic stress response; SAM, sterile-α motif; SFBD, stress fiber-binding domain; ZAK/MAP3K20, mitogen-activated protein kinase kinase 20.

The functional versatility of ZAK arises from a pronounced structural and functional divergence between its two principal splice variants: ZAKα and ZAKβ (Liu et al., 2000; Khani et al., 2022). Both variants share a conserved N-terminal catalytic kinase domain and a leucine-zipper (LZ) motif. Structural studies indicate that this LZ motif enables homodimer formation, facilitates autophosphorylation, and promotes the activation of downstream signaling pathways (Yang, 2002). However, they exhibit marked distinctions in their C-terminal regulatory regions. ZAKα possesses a sterile-α motif (SAM) domain that acts as an essential sentinel for ribotoxic stress, whereas ZAKβ lacks this motif entirely and instead carries a unique stress fiber-binding domain (SFBD) that functions in homeostatic mechanotransduction, as illustrated schematically in Figure 1 and summarized comprehensively in Table 1 (Chang et al., 2016; Lee et al., 2018).

**TABLE 1:** Comparison of structural, physiological, and pathophysiological characteristics between ZAKα and ZAKβ isoforms.

Feature	ZAKα isoform	ZAKβ isoform
C-terminal structure	Characterized by a highly conserved SAM domain located distal to the LZ motif	Lacks the SAM domain entirely; possesses a unique C-terminal SFBD.
Tissue expression profile	Generally low in healthy adult tissues; frequently upregulated in pathological states and malignancies	Constitutively highly expressed in high-energy, contractile tissues (e.g., myocardium, skeletal muscle), as well as in normal colonic epithelium
Primary physiological role	Sentinel for RSR; orchestrates embryonic limb and auditory system development	Specialized mechanosensor; maintains structural and functional integrity of myofibers
Signaling dynamics and interaction	Forms homodimers via the LZ motif to activate downstream MAP2K/MAPK signaling cascades	Formulates heterodimers to attenuate ZAKα signaling in the myocardium; transduces compressive cues in skeletal muscle; exhibits context-dependent synergy with ZAKα in specific malignancies
Pathophysiological implications	Highly context-dependent: Frequently acts as a driver of oncogenesis, cardiac hypertrophy, tissue fibrosis, acute inflammation, and metabolic decline; yet can pivot to function as a tumor suppressor in specific contexts (e.g., HCC)	Dual nature: Exerts potent cardioprotective effects in the myocardium, but can pathogenically contribute to colorectal cancer progression and synergize with ZAKα to induce apoptosis in osteosarcoma treatments
Associated genetic disorders	SHFM accompanied by sensorineural hearing loss	Congenital myopathy with fiber-type disproportion (mild myofibrillar myopathy)

SAM, sterile-α motif; LZ, leucine-zipper; SFBD, stress fiber-binding domain; RSR, ribotoxic stress response; MAP2K, mitogen-activated protein kinase; MAPK, mitogen-activated protein kinase; HCC, hepatocellular carcinoma; SHFM, split-hand/foot malformation.

Despite their divergent C-terminal domains, these two isoforms exhibit profound functional convergence in downstream signaling and are capable of direct coordination through their shared N-terminal architecture. On one hand, ZAK primarily exerts its effects by phosphorylating specific members of the MAP2K family, with a pronounced preference for MKK3/6 (driving p38) and MKK4/7 (driving JNK). These pathways collectively constitute the canonical ZAK-MAPK signaling axis, which subsequently influences ERK signaling and NF-κB transcriptional activity (Zwang et al., 2017). On the other hand, their shared N-terminal LZ motif enables direct physical interaction. Based primarily on *in vitro* expression models, it is proposed that ZAKβ can bind to ZAKα through heterodimerization and promote the downregulation of ZAKα protein levels, thereby acting as an endogenous attenuator of its signaling output (Fu et al., 2016).

Under physiological conditions, ZAKβ predominates in highly contractile, energy-demanding tissues such as cardiac and skeletal muscle, whereas ZAKα expression is relatively low in normal adult tissues but often increases in pathological settings, particularly in malignant diseases (Liu et al., 2014). Functionally, while ZAKα typically activates signaling pathways associated with cell proliferation and pathological tissue remodeling, and ZAKβ acts as an intrinsic negative regulator, their respective roles are largely dependent on the specific cellular environment. This general binary pattern can sometimes be disrupted by specific tissue needs or synergistic interactions. For example, in certain oncology contexts, ZAKα may exhibit potent antitumor properties; in such cases, ZAKβ can synergize with the α subtype to promote rather than inhibit therapeutic apoptosis (Fu et al., 2018a; Xu et al., 2014).

One of the most distinctive functions of ZAK is its role in sensing ribotoxic stress. Specifically, ZAKα detects ribosome stalling and collisions through its specialized C-terminal domain, where the SAM domain facilitates dimerization at the interface of collided ribosomes, thereby initiating the ribotoxic stress response (RSR) (Vind et al., 2026). Furthermore, this activated ZAK signaling has recently been shown to bridge cytoplasmic ribotoxic stress with nuclear DNA repair by phosphorylating the Integrator complex subunit 12 (INTS12) (Li et al., 2026). To fully appreciate this role, it is conceptually crucial to position ZAK accurately within the broader cellular stress surveillance network, particularly distinguishing the ZAK-mediated RSR from the classic Integrated Stress Response (ISR). While the ISR is primarily driven by stress kinases (such as GCN2, PERK, PKR, and HRI) that converge on eIF2α phosphorylation in response to primary stimuli like amino acid deprivation or endoplasmic reticulum (ER) stress (Pak et al., 2016; Mukhopadhyay et al., 2023), the RSR operates through a distinct paradigm. Rather than sensing metabolic deficits directly, ZAKα serves as a highly specialized sensor for the physical collision of stalled ribosomes (Vind et al., 2020b). Thus, instead of acting as a universal stress kinase, ZAK occupies a precise network niche: it bridges structural ribosomal distress with MAPK-dependent signaling cascades, operating parallel to, and occasionally intersecting with, the ISR. Given that excessive activation of this ZAK-driven cascade can be harmful, ZAK signaling is tightly controlled through multiple negative feedback mechanisms, including modulation by the cellular redox environment (Wu et al., 2020; Snieckute et al., 2023).

The extensive signaling capabilities of ZAK underpin a variety of physiological and developmental functions. A fundamental conceptual aspect of ZAK biology is how its distinct expression patterns intrinsically dictate these homeostatic roles. A notable paradox regarding the ZAKα isoform is its remarkably low expression in most normal adult tissues. However, this low basal abundance is an evolutionary requirement rather than an indicator of functional insignificance. Because ZAKα acts as an ultra-sensitive biophysical sensor for the ribotoxic stress response (Snieckute et al., 2023), high constitutive expression in steady-state tissues would lower the activation threshold, risking continuous and aberrant MAPK cascades in response to minor physiological fluctuations. Consequently, its low expression serves as a strict regulatory threshold, maintaining it as an acute stress-response mediator. At the cellular level, this stress-response regulation is evident in proliferating cells, where ZAK governs cell fate at the G2/M checkpoint by orchestrating p38/JNK-dependent phosphorylation cascades, which dictate whether cells temporarily halt the cell cycle for repair or execute apoptosis under severe stress (McKenney et al., 2025; Cerezo et al., 2025). In distinct contrast, the ZAKβ isoform acts as a mechanotransduction regulator constitutively expressed at higher levels in contractile tissues, contributing to the maintenance of muscle fiber structural integrity (Stonadge et al., 2023). At the systemic level, ZAK is also essential for normal embryonic development, particularly in limb formation and auditory system development (Silva et al., 2022; Funk et al., 2020).

Given its central involvement in cellular stress responses, proliferation control, and mechanosensory signaling, dysregulation of ZAK kinase activity has been implicated in a range of human diseases. In the following sections, we critically evaluate recent advances in the structural biology and canonical signaling mechanisms of ZAK. Furthermore, we examine how these unique structural and biochemical properties directly dictate its emerging pathogenic roles across diverse systems, ranging from oncology and cardiovascular remodeling to metabolic decline and inherited myopathies. Crucially, we discuss the growing interest in targeting ZAK as a therapeutic strategy, evaluating the clinical limitations faced by broad-spectrum kinase inhibitors and identifying structure-guided, isoform-specific interventions as a challenging but highly promising future Frontier in ZAK pharmacology.

## 2 Tumors and cancer

Rather than acting as a universal oncogene or tumor suppressor, ZAK functions as a highly plastic signaling hub in cancer biology, where its specific pathogenic or protective output is strictly dictated by the tissue context and tumor microenvironment. In some malignancies, ZAK functions as a tumor-promoting factor, whereas in other settings it can contribute to apoptotic signaling and tumor suppression. This functional duality is influenced by factors such as alternative splicing, tissue-specific expression patterns, and the interaction of ZAK with MAPK and metabolic signaling pathways within the tumor microenvironment.

### 2.1 Oncogenic signaling and tumor progression

Unlike classic autonomous oncogenic drivers, large-scale genomic profiling indicates that high-frequency genomic amplifications or pathogenic somatic mutations of the ZAK gene are relatively rare in solid tumors. Instead, the genetic evidence supporting its causal involvement in malignancies is predominantly characterized by altered expression levels and dysregulated alternative RNA splicing. Operating primarily as a contextual signaling node, ZAK’s specific biological effects, whether promoting metastasis or inducing apoptosis, are strictly regulated by upstream microenvironmental trigger signals, tissue specificity, and transcriptomic dysregulation (Liu et al., 2014; Seshagiri et al., 2012). A prominent mechanism driving this oncogenic shift is the aberrant modulation of alternative RNA splicing. For example, the pre-mRNA processing factor 6 (PRPF6) frequently binds directly to *ZAK* pre-mRNA, promoting the inclusion of the exon encoding the SAM domain and thereby favoring the production of the ZAKα isoform (Adler et al., 2014). Transcriptomic profiling and The Cancer Genome Atlas (TCGA) datasets confirm that this preferential upregulation of ZAKα is broadly evident across multiple solid tumors, including gastric, colon, breast, and bladder cancers (Huan et al., 2024). Furthermore, experimental studies corroborate that this specific isoform shift to ZAKα, but not ZAKβ, equips cells with anchorage-independent growth capabilities and promotes tumor formation in xenograft models (Rey et al., 2016).

Despite lacking tumor-initiating activity, ZAKβ remains highly expressed in normal colon tissue, where it is presumed to support baseline epithelial homeostasis. Moreover, in colorectal cancer both isoforms contribute to disease advancement by activating specific downstream cascades, namely, the ERK, JNK, and p38 signaling pathways, which facilitate epidermal growth factor (EGF)-dependent cell migration. This effect depends on the kinase activity of ZAK and can be suppressed by pharmacological inhibitors (Rey et al., 2016).

**FIGURE 2:**
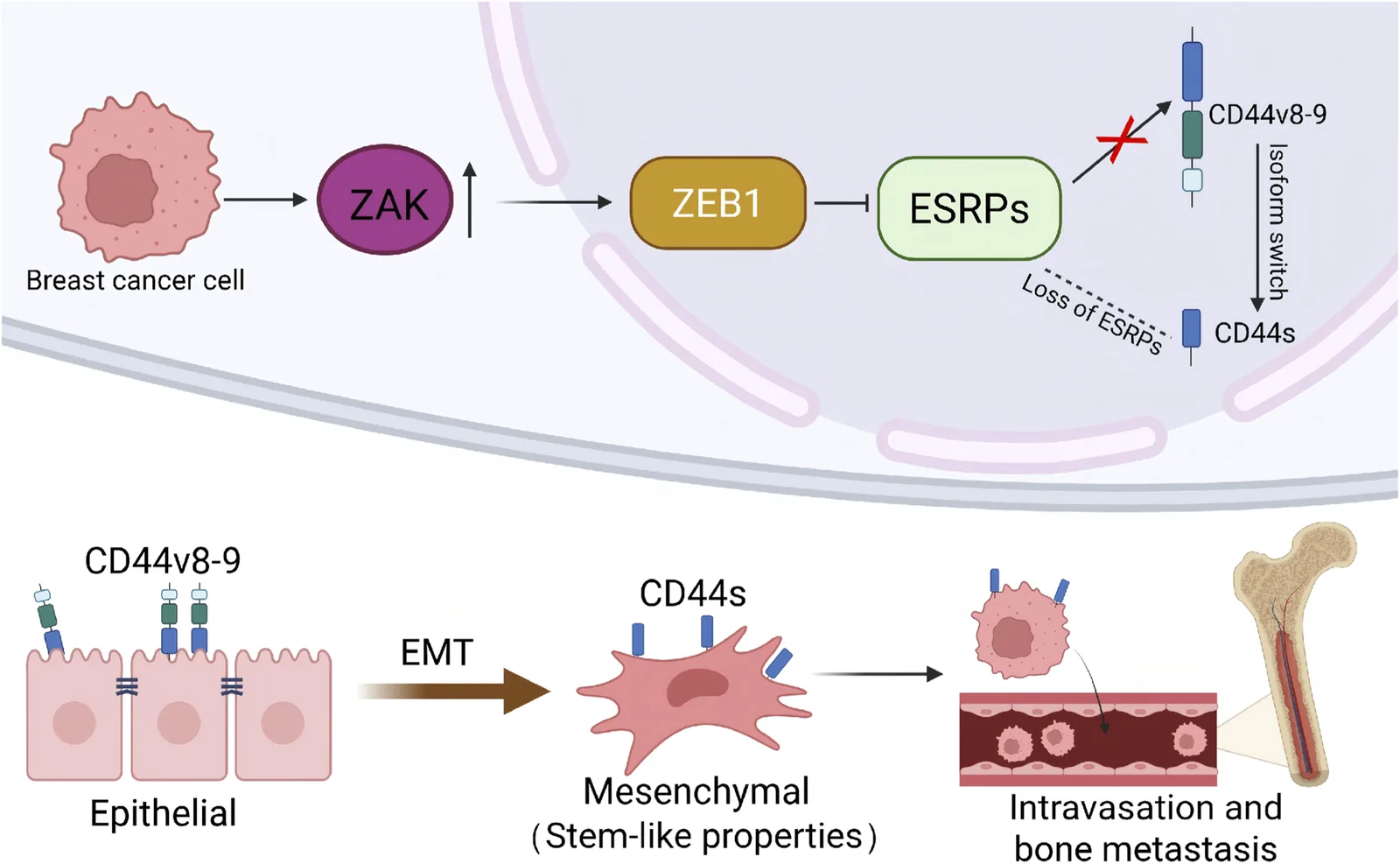
ZAK-mediated pro-metastatic signaling axis in breast cancer. ZAK activates ZEB1 to suppress ESRPs, triggering a pathogenic switch from the epithelial CD44v8-9 to the mesenchymal CD44s isoform, which subsequently induces EMT and promotes bone metastasis. CD44s, standard CD44; CD44v8-9, variant 8–9 of CD44; EMT, epithelial-mesenchymal transition; ESRPs, epithelial splicing regulatory proteins; ZEB1, zinc finger E-box-binding homeobox 1.

Going beyond the enhancement of simple cellular motility, ZAK profoundly drives tumor malignancy by initiating the pathogenic programming of epithelial-mesenchymal transition (EMT). In breast cancer, research by Li et al. (Li et al., 2018) demonstrated that elevated ZAK expression functionally reprograms cells to become highly migratory and metastatic. Mechanistically, increased ZAK promotes the transition from the epithelial CD44v8-9 isoform to the mesenchymal CD44s variant by activating the transcription factor zinc finger E-box-binding homeobox 1 (ZEB1) and suppressing epithelial splicing regulatory proteins (ESRPs). This targeted molecular reprogramming enhances stem-like characteristics and metastatic potential, particularly facilitating bone metastasis (Tolboom et al., 2004), and has led to the identification of ZAK as an independent prognostic indicator of poor clinical outcomes in invasive breast cancer and other malignancies (Figure 2).

Further illustrating its tissue-specific mechanisms, clinical expression profiling in gliomas shows that ZAK expression inversely correlates with patient survival, particularly in low-grade tumors. A recently identified regulatory mechanism involves alterations in the blood-tumor barrier (BTB). In this context, the long noncoding RNA (lncRNA) *MIAT* functions as a competing endogenous RNA that sequesters *miR-140-3p*, thereby relieving its inhibitory effect on ZAK expression. Elevated ZAK activity subsequently promotes phosphorylation of the downstream effector NF-κB p65, leading to transcriptional suppression of tight-junction proteins such as Zonula occludens-1 (ZO-1), occludin, and claudin-5. The resulting reduction in junctional integrity increases BTB permeability and contributes to glioma pathogenesis (He et al., 2020; Duan et al., 2016).

### 2.2 Metabolic reprogramming and DNA sensing

Recent studies increasingly implicate ZAK signaling in the metabolic alterations that support tumor development. *In vitro* studies using lung squamous cell carcinoma (LUSC) cell lines indicate that ZAK participates in a novel, noncanonical pathway triggered by cytosolic DNA detection. Unlike the well-characterized cGAS-STING signaling axis (Chen et al., 2016; Kwon and Bakhoum, 2020), cytoplasmic DNA in LUSC cells preferentially recruits the DNA-dependent protein kinase (DNA-PK) complex. Activation of DNA-PK subsequently stimulates the ZAK-AKT-mTOR signaling cascade (Wang et al., 2024). Crucially, this axis operates as a parallel input to ZAK, entirely distinct from its classical role in the RSR. This bifurcation demonstrates that ZAK does not solely transmit structural ribosomal distress, but functions as a versatile signaling hub capable of translating distinct microenvironmental stressors into highly specific survival or metabolic outputs.

However, it is critical to note that current studies defining this signaling have primarily utilized pan-ZAK experimental approaches (i.e., methods that simultaneously target both ZAKα and ZAKβ indiscriminately). Because the activating phosphorylation site (Thr169) is conserved across both major splice variants, it remains conclusively unresolved whether this DNA-PK-driven activation is strictly isoform-specific or represents a shared kinase capability. Regardless of the specific isoform involved, this cascade enhances glycolytic activity, thereby supplying the energy and biosynthetic intermediates required for tumor growth and contributing to the development of chemotherapy resistance—highlighting a major paradigm shift where a classical MAP3K functionally crosses over to critically modulate AKT/mTOR-driven metabolic rewiring.

**FIGURE 3:**
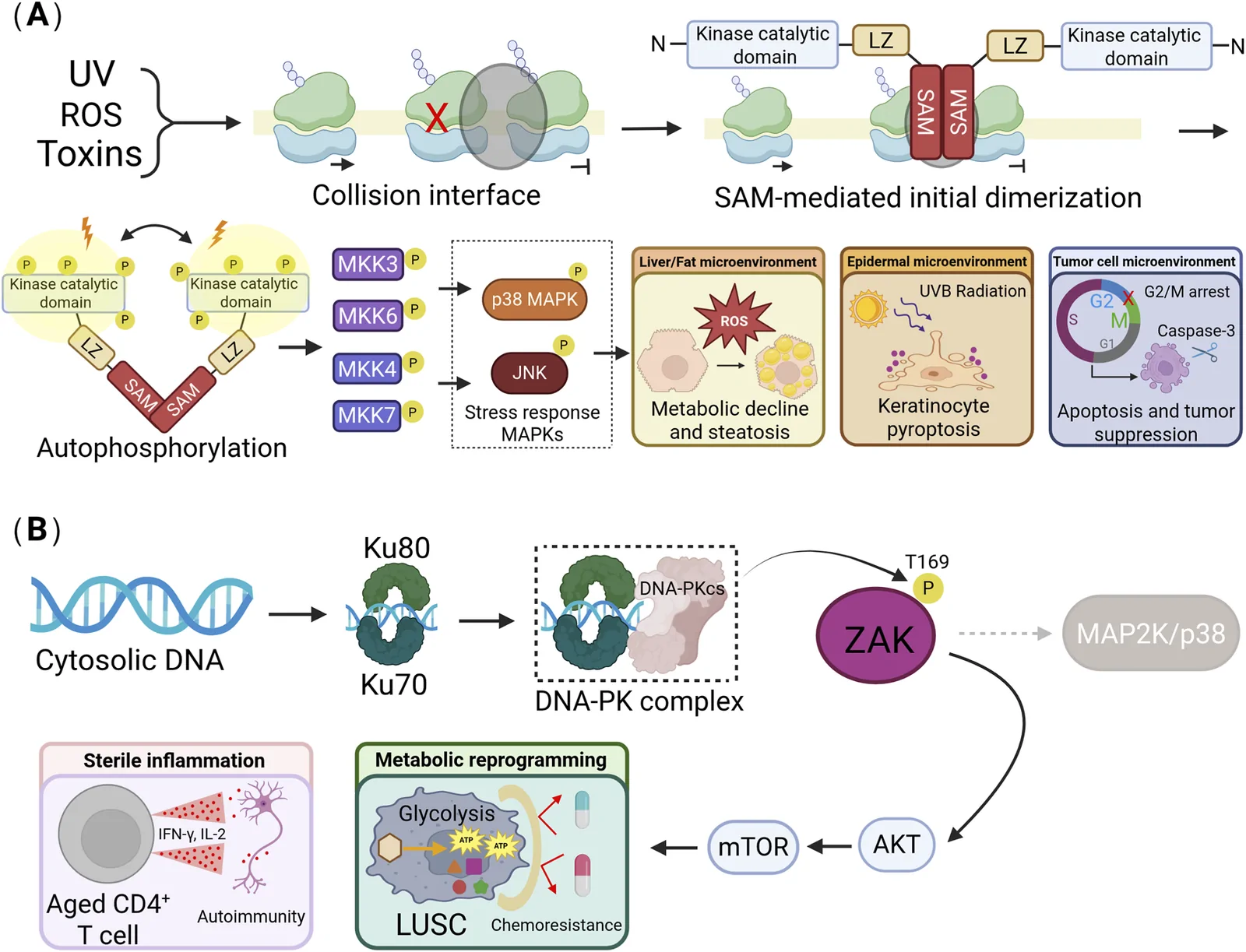
Molecular signaling cascades and tissue-specific pathobiology of ZAK. **(A)** The RSR pathway. ZAKα senses ribosome collisions induced by exogenous stressors (e.g., UV, ROS, toxins). This triggers a MAP3K-MAP2K-MAPK cascade (activating p38/JNK) that drives tissue-specific outcomes, including metabolic decline, epidermal pyroptosis, and tumor apoptosis. **(B)** Non-canonical cytosolic DNA-sensing pathway. Cytosolic DNA activates the DNA-PK complex (Ku70/80 and DNA-PKcs), which phosphorylates ZAK (T169) to engage the AKT/mTOR axis. Dysregulation of this cascade fuels metabolic reprogramming in LUSC and autoimmunity in aged CD4^+^ T cells. AKT, protein kinase B; DNA-PK, DNA-dependent protein kinase; DNA-PKcs, DNA-dependent protein kinase catalytic subunit; IFN-γ, interferon-γ; IL-2, interleukin-2; JNK, c-Jun N-terminal kinase; Ku70/80, Lupus Ku autoantigen protein p70/p80; LUSC, lung squamous cell carcinoma; LZ, leucine-zipper; MAPK, mitogen-activated protein kinase; MKK, MAP kinase (member of the MAP2K family); mTOR, mammalian target of rapamycin; ROS, reactive oxygen species; RSR, ribotoxic stress response; SAM, sterile-α motif; T169, threonine 169; UV, ultraviolet.

Beyond metabolic control, functional assays including wound healing and Transwell studies have demonstrated that the depletion of ZAK markedly impairs the migratory and metastatic potential of LUSC cells (Wang et al., 2024). Given that evidence in this domain is currently limited to a few foundational studies, the intricate crosstalk between ZAK, tumor metabolism, and metastasis remains an emerging area requiring further investigation. The classical ZAK-mediated RSR (Figure 3A), along with the detailed mechanisms of this DNA-sensing network (Figure 3B), are summarized here.

### 2.3 Tumor suppressive functions and apoptosis

Despite its documented oncogenic activities in certain cancers, ZAK functions as a key mediator of stress-induced apoptotic responses in specific cellular contexts. Early cloning and expression studies demonstrated that the ZAK protein contains domains capable of driving cell arrest and apoptosis. Consistent with this, in hepatocellular carcinoma (HCC) models where the ZAKα isoform is frequently suppressed by the lncRNA *URHC*, the restoration of ZAKα expression activates the ERK/MAPK signaling pathway, inducing G0/G1 phase arrest and terminal cell death (Xu et al., 2014). Similarly, in non-small cell lung cancer (NSCLC), ZAK suppresses tumor cell proliferation through the ERK/JNK/AP-1 signaling axis. As expected, targeted knockdown of ZAK in these cultured models readily abolishes the cell-cycle blockade and accelerates *in vitro* tumor growth (Xu et al., 2014; Yang et al., 2010).

Interestingly, the function of ZAK under exogenous therapeutic stress is highly pleiotropic and can paradoxically shift toward cell survival. For instance, in *PIK3CA*-mutant breast cancer cells, shRNA-mediated silencing of ZAK synergizes with PI3K inhibitors to induce massive cell death, indicating its role as an essential survival gene under specific therapeutic pressures (Zwang et al., 2017). Furthermore, silencing ZAK dramatically sensitizes cultured medulloblastoma cells to ionizing radiation (Markowitz et al., 2016).

Conversely, in other therapeutic paradigms, ZAK acts as a critical executioner of drug-induced cytotoxicity via the RSR. In osteosarcoma models, exposure to doxorubicin causes a significant upregulation of ZAKα, which is absolutely required for the downstream activation of caspase-3 and subsequent apoptosis (Fu et al., 2018b). Recent breakthroughs in chronic myeloid leukemia (CML) demonstrate that targeted tyrosine kinase inhibitors (TKIs) actively provoke structural ribosome collisions. Rather than acting through classical kinase inhibition, these TKIs implicitly rely on the consequent ZAK-dependent p38 activation to effectively trigger CML cell lethality (Park et al., 2026).

Further complicating this paradigm, the canonical antagonism between ZAK isoforms is inverted in certain oncology contexts. Also in osteosarcoma, ZAKβ synergizes with ZAKα to drastically amplify apoptotic programs (Fu et al., 2018a). Treatment with flavonoid compounds such as 3-hydroxy-2-phenylchromone (3-HF) selectively increases ZAKβ expression, which subsequently elevates ZAKα levels to activate JNK-dependent mitochondrial apoptosis (Fu et al., 2020). Additional natural compounds also modulate ZAK-associated signaling pathways to trigger cytotoxic effects. Fisetin and BA-TPQ, for instance, induce cancer cell death through activation of the Hippo signaling pathway and the JNK/TGF-β signaling cascade, respectively. It is important to distinguish that this specific cytotoxic mechanism in cancer cells operates distinctly from the pathogenic TGF-β cascades that drive structural remodeling in the myocardium (Chen et al., 2013; Fu et al., 2019).

## 3 Cardiovascular diseases and cardiomyopathy

Although ZAK is widely studied in oncology, its high constitutive expression in metabolically demanding tissues underscores its importance in cardiovascular biology. The heart, which requires tightly regulated stress-response signaling, relies on ZAK-mediated pathways to maintain structural and functional integrity under normal physiological conditions. However, disruption of ZAK signaling has therefore been linked to several cardiac pathologies, including cardiac hypertrophy, myocardial fibrosis, and cardiotoxicity associated with chemotherapy.

### 3.1 ZAK as a mediator of cardiac hypertrophy

Evaluating the role of ZAK in hypertrophic cardiomyopathy (HCM) requires distinguishing between clinical observations and specific experimental models. While causal genetic mutations in ZAK are not classically associated with HCM, its clinical relevance is firmly established through significant altered expression profiles. Current evidence is derived primarily from *in vitro* cultured cardiomyoblasts (e.g., H9c2 cells) and supported by expression analyses in human cardiac tissue (Lin et al., 2022; Pai et al., 2018).

Strong evidence establishes ZAK as a pathogenic amplifier driving the progression of HCM. In human cardiac tissue, rather than initiating the pathology, ZAKα detects primary pathological stress signals and amplifies them into sustained, high-amplitude activation of these classical MAPK cascades, thereby driving a hypertrophic program characterized by enlargement of cardiomyocytes and increased expression of classical hypertrophy-associated markers such as atrial natriuretic factor (ANF) and brain natriuretic peptide (BNP) (Yang et al., 2020). The initiation of this hypertrophic response depends on ZAK homodimerization mediated by the LZ domain, followed by autophosphorylation. Once activated, the kinase preferentially directs these signals to the nuclear accumulation and activation of the transcription factors GATA4 and c-Jun (Pai et al., 2018). Importantly, this hypertrophic remodeling occurs independently of cell-cycle progression, even though p21^Waf1/Cip1^ expression increases concurrently, and does not involve the classical ERK or calcineurin-NFATc3 signaling pathways (Hsieh et al., 2015; Huang et al., 2004a).

Expanding beyond these classical kinase cascades, ZAK orchestrates this structural reprogramming through a highly interconnected signaling network. Beyond classical MAPKs, it acts as a critical downstream effector for pro-hypertrophic stimuli like TGF-β. Following TGF-β stimulation, ZAK interacts directly with TGF-β receptor I, enhancing receptor autophosphorylation and facilitating the recruitment of MKK7, which subsequently promotes further *ANF* gene transcription (Huang et al., 2004b). To prevent unchecked structural alterations, this amplification system is fine-tuned by intrinsic regulatory loops. For example, while Rho GDP-dissociation inhibitor beta (RhoGDIβ) typically stimulates Rac1-dependent pathways to promote hypertrophic growth and cell migration, ZAK can directly bind and phosphorylate RhoGDIβ, thereby exerting a negative feedback counterbalance (Huang et al., 2009a; Huang et al., 2009b).

While these molecular interactions elegantly delineate the pro-hypertrophic signaling axes of ZAKα *in vitro*, a notable gap remains in the literature. Further robust *in vivo* animal models and human clinical data are required to fully map out how these precise feedback mechanisms operate dynamically within the intact failing myocardium.

### 3.2 Myocardial fibrosis and extracellular matrix remodeling

Operating in parallel with cellular hypertrophy, clinical observations have detected elevated ZAK expression in approximately 80% of human myocardial scar tissues, underscoring its potential role in the development of cardiac fibrosis and extracellular matrix (ECM) dysregulation. Following cardiac injury or chronic stress, ZAK disrupts the balance of ECM turnover by altering the secretory profile of myocardial cells (Travers et al., 2016; Rose et al., 2010). First, it directly stimulates the expression of tissue inhibitors of metalloproteinases (TIMP-1 and TIMP-2), which suppress the activity of matrix metalloproteinase-9 (MMP-9). Concurrently, ZAK maintains persistent downstream MAPK signaling, resulting in enhanced MMP-2 activity (Cheng et al., 2009).

This dysregulated turnover accelerates the accumulation of fibrotic matrix components, ultimately contributing to structural remodeling and mechanical dysfunction of the failing myocardium (Perestrelo et al., 2021; Frangogiannis, 2019). While these cardiomyocyte-driven alterations clearly influence the local ECM, direct evidence defining ZAK’s intrinsic role within primary cardiac fibroblasts remains sparse. Whether ZAK autonomously promotes fibroblast activation or solely mediates profibrotic signaling downstream of stressed cardiomyocytes represents a critical emerging area that requires further *in vivo* and cell-type-specific investigation.

### 3.3 Isoform-specific antagonism and protective mechanisms

While ZAK broadly exhibits profound context-dependency across diverse tissues, its biological functions within the specialized cardiac microenvironment are predominantly governed by the strict functional opposition between its two major isoforms. In the context of cardiac stress, ZAKα predominantly orchestrates detrimental cardiac alterations. Highlighting its transcriptomic dysregulation in human pathology, its expression is markedly upregulated in disease states such as myocardial infarction, diabetic cardiomyopathy, and age-related cardiac remodeling (Wang et al., 2016). In these settings, sustained activation of ZAKα drives MAPK signaling, which promotes hypertrophy and initiates caspase-3-dependent apoptotic pathways, including both Fas-mediated and mitochondrial mechanisms. Specifically, this robust ZAKα-MAPK output upregulates the expression of death receptors and their ligands (e.g., Fas/FasL) to trigger the extrinsic apoptotic cascade. Concurrently, these stress kinases modulate Bcl-2 family proteins to induce mitochondrial outer membrane permeabilization, leading to cytochrome c release and the subsequent intrinsic activation of the caspase cascade (Fu et al., 2016).

*In vitro* studies using cultured cardiomyoblasts suggest that ZAKβ may function as an endogenous cardioprotective regulator. As previously noted, ZAKβ directly modulates the magnitude of ZAKα signaling via heterodimerization, thereby serving as an intrinsic molecular brake to prevent excessive activation of the pro-apoptotic cascade under cardiac stress (Fu et al., 2016). Although this mechanism is supported by experimental data, additional *in vivo* studies in mature cardiomyocytes are required to fully establish the protective role of ZAKβ in human cardiac physiology (Branco et al., 2015; Kuznetsov et al., 2015).

**FIGURE 4:**
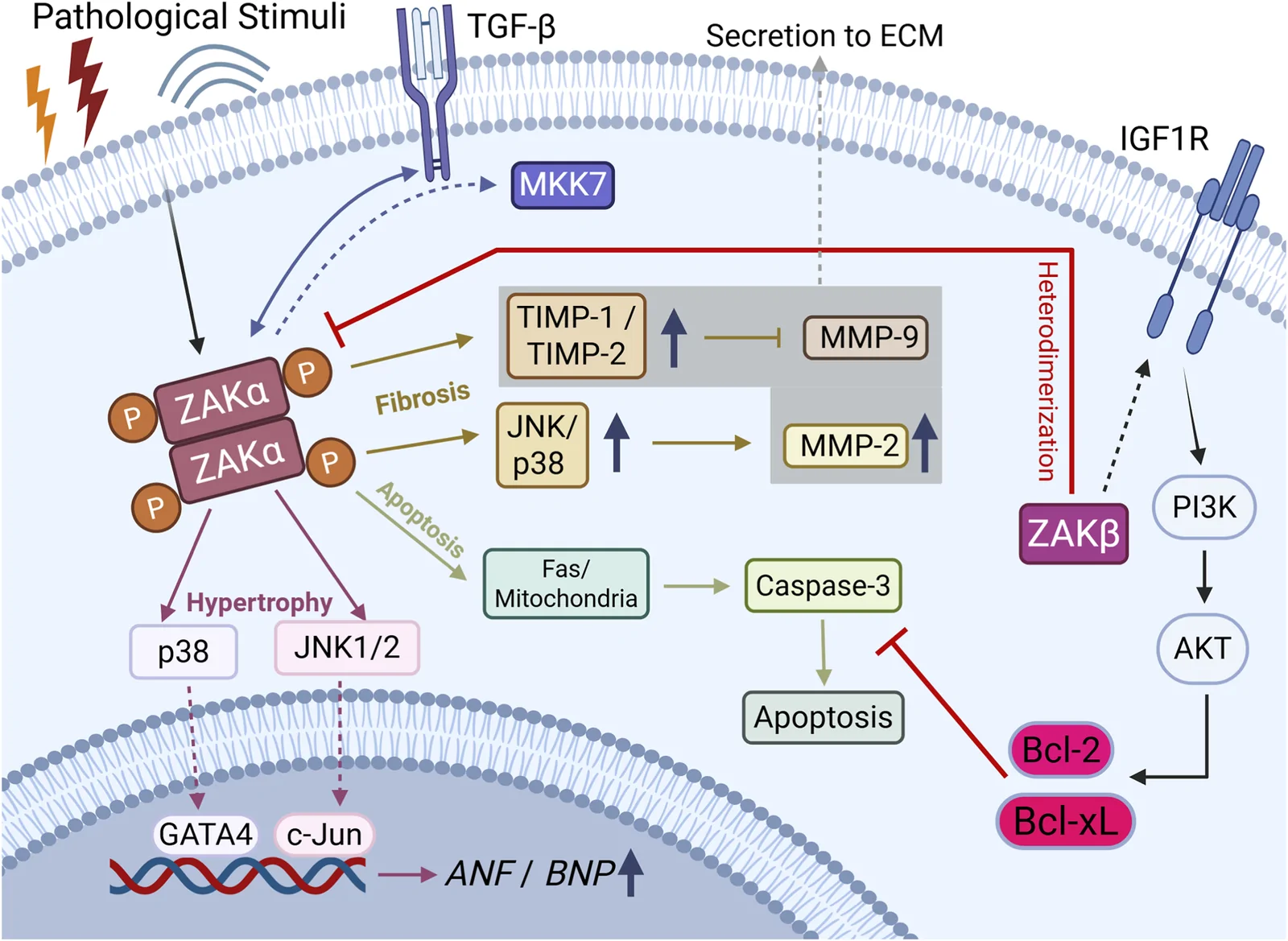
Molecular mechanisms of ZAK-mediated cardiac remodeling and isoform-specific cardioprotection. Pathological stimuli activate ZAKα homodimers, which drive cardiac hypertrophy (via p38/JNK-mediated transcription), fibrosis (via ECM remodeling), and apoptosis. Conversely, ZAKβ confers cardioprotection through a dual mechanism: it directly antagonizes ZAKα via heterodimerization and independently promotes cell survival by activating the IGF1R/PI3K/AKT signaling axis. AKT, protein kinase B; ANF, atrial natriuretic factor; Bcl-2, B-cell lymphoma 2; Bcl-xL, B-cell lymphoma-extra large; BNP, brain natriuretic peptide; ECM, extracellular matrix; IGF1R, insulin-like growth factor 1 receptor; JNK, c-Jun N-terminal kinase (specifically labeled as JNK1/2 in the schematic to denote the principal functional isoforms); MKK, MAP kinase (member of the MAP2K family); MMP, matrix metalloproteinase; PI3K, phosphoinositide 3-kinase; TGF-β, transforming growth factor-beta; TIMP, tissue inhibitor of metalloproteinases.

Beyond this inhibitory interaction, ZAKβ also supports cardiomyocyte survival through independent signaling axes. Specifically, ZAKβ can activate the IGF1R/PI3K/AKT signaling cascade, leading to increased expression of anti-apoptotic proteins such as Bcl-2 and Bcl-xL (Raja Singh et al., 2014; Chen and Sun, 2012). The protective effects of ZAKβ are particularly evident under conditions of oxidative stress. In experimental models, ZAKβ overexpression significantly reduces oxidized low-density lipoprotein (ox-LDL)-induced apoptosis and hypertrophic responses in cardiomyocytes (Lin et al., 2022; Olejnik et al., 2023). Taken together, the precise stoichiometric balance between the opposing ZAKα and ZAKβ isoforms does not merely modulate stress signals, but functions as a biochemical switch dictating the ultimate survival or decompensation of the myocardium under pathological stress (Figure 4).

## 4 Diverse pathologies: Stratifying evidence from human genetics to experimental models

In addition to its roles in cancer and cardiovascular disease, ZAK has recently been identified as a central regulator of various physiological and pathological processes. The functional divergence between ZAKα and ZAKβ contributes to its involvement in metabolic dysfunction, congenital abnormalities, autoimmune conditions, and fibrotic diseases. In this section, we stratify the diverse pathological contexts of ZAK signaling based on the quality of the underlying evidence, categorizing them into direct human genetic disorders and emerging experimental disease models. To clearly delineate its causal and clinical relevance, Table 2 comprehensively summarizes the diverse genetic, genomic, and transcriptomic evidence supporting ZAK’s involvement across these human pathologies, ranging from causal mutations to profound altered expression profiles.

**TABLE 2:** Summary of genetic and transcriptomic evidence for ZAK involvement in pathologies.

Disease/Pathology	Type of evidence	Specific alterations and clinical observations
Congenital myopathy	Genetic mutations	Recessive loss-of-function mutations (frameshift and nonsense variants) resulting in the ablation of the SFBD.
Split-hand/Foot malformation (SHFM)	Genetic mutations	Autosomal recessive missense variants (e.g., p.Phe368Cys) physically disrupting the SAM domain
Solid tumors (gastric, colon, breast, bladder)	Transcriptomic dysregulation and altered splicing	High-frequency aberrant alternative splicing (mediated by PRPF6) driving preferential ZAKα upregulation; evident in TCGA cohorts
Glioma	Altered expression	Expression inversely correlates with patient survival; ceRNA mechanism by lncRNA *MIAT*.
Cardiac fibrosis and hypertrophy	Altered expression	Elevated ZAK expression detected in ∼80% of human myocardial scar tissues; markedly upregulated in myocardial infarction and diabetic cardiomyopathy
Metabolic decline and obesity	Genomic ablation (experimental)	Genomic deletion of *Zak* in murine models confers profound resistance to high-fat/high-sugar diet-induced hepatic steatosis
Bacterial infections	Altered expression	Dynamic modulations in expression during *Helicobacter pylori* colonization and microRNA-mediated suppression during *Salmonella* infection

ceRNA, competing endogenous RNA; lncRNA, long noncoding RNA; PRPF6, pre-mRNA, processing factor 6; SAM, sterile-α motif; SFBD, stress fiber-binding domain; SHFM, split-hand/foot malformation; TCGA, the cancer genome atlas; ZAK, mitogen-activated protein kinase kinase 20.

### 4.1 High-confidence evidence: The structural basis of ZAK mutations in human genetic and developmental disorders

#### 4.1.1 Skeletal muscle physiology and congenital myopathies

In striking contrast to its reactive, stress-amplifying role in systemic pathologies, ZAK acts as a primary, cell-autonomous disease driver within specific hereditary contexts. This primary pathogenic role is most clearly illustrated in skeletal muscle physiology, where the ZAKβ isoform is indispensable for maintaining myofibrillar structure and homeostatic function (Stonadge et al., 2023).

In skeletal tissue, ZAKβ operates as a specialized mechanosensory signaling molecule that directly responds to mechanical stress generated during muscle contraction. This activation occurs via the characteristic SFBD, which physically tethers to the Z-disc and actin stress fibers to convert compressive forces and osmotic shock into protective downstream p38 and JNK MAPK cascades (Nordgaard et al., 2022). Studies in human cultured cells, such as U2OS models for actin stress fiber dynamics, reveal a striking mechanosensory specificity: ZAKβ is robustly stimulated by cellular compression but remains unresponsive to mechanical stretch or tension, which is instead monitored by parallel pathways such as TAK1. Furthermore, because ZAKβ physically associates with actin stress fibers, its excessive activation can drive the disassembly of the actin cytoskeleton, leading to dramatic alterations in cell shape and morphological remodeling (Gotoh et al., 2001).

Consequently, genetic disruption of this primary mechanotransduction pathway leads to progressive hereditary myopathies. Providing direct genetic evidence for its causal relevance in human disease, clinical exome sequencing has identified recessive loss-of-function mutations in the ZAK gene, including frameshift and nonsense variants, as a novel genetic cause of congenital myopathy characterized by fiber-type disproportion (Vasli et al., 2017). Individuals harboring these mutations typically exhibit generalized muscle weakness, progressive skeletal deformities such as scoliosis, and impaired respiratory function. Structurally, these truncating variants completely ablate the C-terminal SFBD. Lacking this crucial domain, the truncated kinase cannot anchor to the Z-disc, thereby abolishing its localized mechanosensory capacity. Rather than primarily impairing myoblast fusion, this uncoupling profoundly disrupts the adaptive turnover of structural proteins during mechanical stress. Ultimately, the loss of this localized signaling drives the pathological mislocalization and accumulation of unassembled cytoskeletal components, most notably filamin C (FLNC) and Bcl-2-associated athanogene 3 (BAG3), serving as the central mechanism underlying ZAK-deficient myofibrillar myopathy (Stonadge et al., 2023).

#### 4.1.2 Hereditary developmental anomalies: Split-hand/foot malformation

Despite its characteristically low basal expression in adult tissues, the ZAKα isoform plays a pivotal role in regulating embryonic development, particularly in the formation of limb structures and the auditory system. Similarly, establishing the genomic basis for its role in embryonic development, genetic studies have identified autosomal recessive mutations (such as missense variants) affecting the functionally critical SAM domain of ZAKα as causative factors in Split-Hand/Foot Malformation (SHFM).

At the structural level, clinical ZAK variants exhibit distinct localizations and molecular consequences. While the rare variant p. Ala505Ser resides in a C-terminal region of unknown function, the well-established pathogenic mutation p. Phe368Cys strictly localizes to the SAM domain. Rather than affecting the N-terminal kinase catalytic pocket, the p. Phe368Cys substitution physically disrupts the hydrophobic core of the SAM domain, causing a ∼30% loss of alpha-helicity and promoting protein aggregation (Funk et al., 2020; Spielmann et al., 2016). This severe structural destabilization impairs the developmental function of ZAKα.

At the mechanistic level, loss of SAM domain function leads to a 60% downregulation of *Trp63*, a master transcription factor in ectodermal development (Kataoka et al., 2018). In the context of limb morphogenesis, *Trp63* is required for the stratification and maintenance of the apical ectodermal ridge (AER) (Vernersson Lindahl et al., 2013; Duijf et al., 2003). By regulating *Trp63* expression, ZAKα indirectly sustains the critical AER signaling centers that dictate proper limb outgrowth, cell survival, and digit patterning (Spielmann et al., 2016). This mechanism elegantly explains the variable limb phenotypes observed in patients and animal models with ZAKα SAM domain mutations.

Evidence supporting this pathogenic mechanism has been reproduced in CRISPR/Cas9-based mouse models. While complete systemic deletion of the *ZAK* gene results in embryonic lethality, targeted removal of the SAM domain alone produces viable mice exhibiting complex hindlimb malformations and auditory deficits (Farooq et al., 2020). These *in vivo* findings establish ZAK as an evolutionarily conserved kinase required for proper developmental signaling during mammalian embryogenesis.

### 4.2 Emerging evidence: ZAK as a regulatory hub in systemic pathologies

#### 4.2.1 Metabolic deregulation and aging-related decline

When transitioning from rare monogenic disorders to common, multifactorial human conditions, ZAK predominantly shifts its role from a primary disease initiator to a reactive amplifier of chronic environmental and metabolic stress. Far from being speculative, recent functional genomic and metabolomic investigations have definitively established ZAKα as a systemic metabolic sensor that coordinates cellular responses to nutrient availability (Snieckute et al., 2023; Snieckute et al., 2022).

During severe nutrient stress, such as amino acid deprivation, uncharged tRNAs typically trigger the GCN2-dependent ISR (Costa-Mattioli and Walter, 2020). Concurrently, the stalling of translating ribosomes induces physical collisions that specifically activate the RSR. In this context, ZAKα does not act in isolation; it engages in dynamic crosstalk with broader metabolic kinase networks, including the AMPK and mTOR signaling hubs (Hotamisligil and Davis, 2016). By intersecting with these established networks, the ZAKα-driven RSR helps fine-tune translational control and promotes the secretion of adaptive hormones like fibroblast growth factor 21 (FGF21). This positions ZAKα as an essential parallel amplifier in nutrient sensing, complementing rather than duplicating the canonical ISR pathways (Hotamisligil and Davis, 2016). In pathological conditions such as obesity and biological aging, this adaptive sensing mechanism becomes chronically dysregulated. Elevated levels of intracellular reactive oxygen species (ROS) continuously stimulate ZAKα-dependent RSR signaling. The sustained activation of these stress-responsive MAPKs directly drives metabolic deterioration, which clinically manifests as glucose intolerance and hepatic steatosis (Snieckute et al., 2023; Vind et al., 2025; Li et al., 2025).

The most compelling genetic evidence for this metabolic axis comes from *in vivo* genomic ablation models. When subjected to high-fat and high-sugar diets, ZAK-deficient mice maintain a dramatically protected, lean phenotype. Compared to wild-type controls, they exhibit reduced adipocyte size, increased browning of white adipose tissue, and approximately 50% lower hepatic triglyceride levels (Zhang et al., 2025). Because these murine models robustly recapitulate the pathological trajectory of human obesity and non-alcoholic steatohepatitis (NASH), such profound resistance to metabolic deterioration provides a robust preclinical rationale. Ultimately, these findings highlight that targeted suppression of ZAKα is not merely a theoretical concept, but a highly viable clinical strategy to combat obesity-associated morbidities.

#### 4.2.2 UV radiation, inflammation, and host-pathogen interactions

Parallel to its involvement in systemic metabolic decline, the hyperactivation of ZAK signaling under sustained intracellular stress functions as a critical driver of chronic inflammation and tissue barrier disruption. This pathogenic amplification is particularly evident in immune aging, driven by the noncanonical DNA-PK/ZAK/AKT/mTOR metabolic axis, which promotes excessive CD4^+^ T cell activation and autoimmune inflammation. At the molecular level, the recognition of cytosolic DNA by the Ku70/80 complex recruits the DNA-dependent protein kinase catalytic subunit (DNA-PKcs) (Tang et al., 2023), which subsequently phosphorylates ZAK at its highly conserved Thr169 residue. While the specific ZAK isoform mediating this process remains unresolved, this phosphorylation event significantly potentiates AKT-mTOR signaling. Consequently, it promotes excessive CD4^+^ T cell proliferation and enhanced secretion of pro-inflammatory cytokines such as IFN-γ and IL-2, directly exacerbating age-associated autoimmune diseases like experimental autoimmune encephalomyelitis (EAE) (Chi, 2012; Wang et al., 2021). Recent studies have begun to demonstrate the pathological role of this pathway *in vivo* using T cell-specific ZAK knockout mice (Ye et al., 2023); however, which ZAK splicing isoform mediates this non-canonical axis in immunosenescence still needs to be further elucidated using isoform-specific *in vivo* models.

Beyond endogenous immune senescence, ZAKα serves as an equally vital sentinel and inflammatory mediator when barriers are breached by exogenous insults, such as ultraviolet radiation (Bernard et al., 2012). Contrary to the long-standing view that DNA damage alone drives sunburn pathology, recent landmark studies by Vind et al. and Sinha et al. demonstrate that UVB radiation directly induces physical ribosomal collisions, which specifically activate the ZAKα-mediated RSR. This targeted activation drives downstream MAPK signaling that triggers keratinocyte pyroptosis and apoptosis, alongside significant epidermal hyperplasia, conclusively establishing ZAK as a primary molecular driver of photodamage and acute inflammatory skin disorders (Vind et al., 2024; Sinha et al., 2024).

In the context of host-pathogen interactions, ZAK operates as a highly responsive, and sometimes exploitable, node within the innate immune system. Ribosome-inactivating toxins such as Shiga toxin (Stx) and ricin strongly activate ZAKα/β signaling, which forcefully stimulates IL-8 production and induces apoptosis in intestinal epithelial cells, exacerbating mucosal injury (Stone et al., 2012; Jandhyala et al., 2008). In addition, dynamic modulations in ZAK expression occur during direct bacterial infections, such as *Helicobacter pylori* colonization in gastric tissue (Khani et al., 2022). Similarly, during *Salmonella*-induced necrotic enteritis, host microRNAs (e.g., *gga-miR-200a-3p*) suppress ZAK expression to restrain downstream MAPK-driven inflammatory responses (Pham et al., 2020). Given its capacity to initiate such potent immune signaling, ZAK likely plays a much broader role in systemic antibacterial or antiviral defenses. However, beyond these isolated pathogen and toxin models, the exact scope of its immunological functions during live infections remains largely uncharted territory, highlighting a critical need for future *in vivo* studies utilizing ZAK knockout models.

#### 4.2.3 Tissue fibrosis and stem cell plasticity

Beyond its established impacts on cardiovascular remodeling, ZAK actively orchestrates fibrotic progression in other organ systems, most notably in the pathogenesis of renal tubulointerstitial fibrosis (TIF). Consistent with this, experimental models of chronic kidney disease (CKD) demonstrate distinct altered expression profiles, where ZAK expression is markedly elevated in renal tubular epithelial cells, driving pathogenic progression. In this context, rather than acting purely as a transient stress sensor, ZAK directly enhances signaling through the canonical TGF-β/Smad2/3 pathway, thereby driving the pathological deposition of extracellular matrix and exacerbating tissue scarring (Shu et al., 2022).

Crucially, the regulatory influence of ZAK extends far beyond extracellular matrix deposition; it operates as a pivotal biochemical rheostat governing cellular plasticity within epithelial tissues under environmental stress. This capacity is profoundly illustrated within the intestinal epithelium during nutrient deprivation, where basal ZAKα signaling serves as a critical stress-response threshold. Under severe amino acid restriction, the activation of ZAKα initiates a noncanonical proto-oncogene tyrosine-protein kinase (SRC)-Yes-associated protein (YAP) signaling axis. This axis operates entirely independently of the traditional MAPK or GCN2 stress-response pathways. Instead of promoting differentiation or altering the microenvironment, this unique signaling detour directly acts on *Lgr5* ^+^ intestinal stem cells (ISCs), forcing them to abandon their mature stemness program and regress into a highly plastic, fetal-like state (Silva et al., 2022; Gregorieff et al., 2015; Guillermin et al., 2021). This adaptive regression appears to be essential for preserving the ISC pool during mucosal injury and promoting fibrotic remodeling, underscoring the context-specific versatility of ZAK and suggesting promising therapeutic relevance for intestinal regeneration and inflammatory bowel disease.

## 5 Therapeutic targeting and future outlook

Because ZAK acts as a key regulator of both ribotoxic stress signaling and mechanically induced pathways, it offers promise as an attractive, albeit complex, therapeutic target. Effective pharmacological modulation of ZAK must account for its distinct physiological roles across tissues as well as the functional opposition between the ZAKα and ZAKβ isoforms. A summary of currently identified pharmacological agents capable of modulating ZAK signaling, together with their potential clinical applications and associated limitations, is presented in Table 3.

**TABLE 3:** Summary of pharmacological modulators targeting ZAK and their clinical or experimental implications.

Pharmacological agent	Classification	Developmental stage	Mechanism of action/Target specificity	Therapeutic potential/Application	Toxicities and clinical limitations
Nilotinib/Ponatinib/Sorafenib/Regorafenib	Multi-kinase inhibitors	Clinical (FDA-approved for other indications)	Exhibit potent off-target anti-ZAK activity; block doxorubicin-triggered MAPK signaling	Repurposed to prevent anthracycline-induced cardiotoxicity	Broad-spectrum inhibition limits systemic application (specific ZAK-related DLTs not applicable due to off-target nature)
PLX4720	Off-target ZAK inhibitor	Preclinical (in ZAK contexts)	Direct off-target ZAK inhibition	Promotes apical extrusion and elimination of transformed epithelial cells	Highly tissue-context dependent
Vemurafenib	BRAF inhibitor	Clinical (FDA-approved)	Binds the ZAK kinase pocket with high affinity; blocks JNK-mediated apoptotic pathway in the epidermis	Melanoma treatment	Severe “on-target, off-tissue” toxicity: Allows UV-damaged keratinocytes to proliferate, driving cSCC
iZAK2	Specific ZAK inhibitor	Preclinical	Dismantles the non-canonical DNA-PK/ZAK/AKT/mTOR metabolic axis	High efficacy in suppressing LUSC tumor growth	Clinical outcomes and dose-limiting toxicities have not yet been evaluated in humans
Covalent inhibitors	Novel structure-based inhibitors	Preclinical	Selectively target the unique Cys22 residue within the ZAK ATP-binding pocket	Nanomolar potency for irreversible blockade of ZAK-mediated signaling	Undetermined in humans
Compound 3h	ZAK-specific antagonist	Preclinical	Exceptional selectivity for ZAK over other MAP3Ks	Dramatically alleviates left ventricular hypertrophy and myocardial disarray (oral administration)	Undetermined in humans
Compound 6p	ZAK-specific antagonist	Preclinical	Attenuates ZAK-driven ECM remodeling	Profoundly alleviates ECM deposition in ischemic and obstructive kidney injury models	Undetermined in humans

MAPK, mitogen-activated protein kinase; BRAF, B-Raf proto-oncogene, serine/threonine kinase; JNK, c-Jun N-terminal kinase; UV, ultraviolet; cSCC, cutaneous squamous cell carcinoma; DNA-PK, DNA-dependent protein kinase; AKT, protein kinase B; mTOR, mammalian target of rapamycin; LUSC, lung squamous cell carcinoma; Cys22, cysteine-22; ATP, adenosine triphosphate; ECM, extracellular matrix.

### 5.1 Repurposing of kinase inhibitors and the toxicity paradox

Historically, inhibition of ZAK activity has occurred inadvertently through the use of various clinically approved kinase inhibitors developed for other therapeutic indications. Several clinically approved agents, including nilotinib, ponatinib, sorafenib and regorafenib, exhibit substantial off-target inhibitory activity against ZAK (93). In certain contexts, this broad-spectrum inhibition presents a localized therapeutic opportunity. For example, compounds such as nilotinib and sorafenib have been experimentally repurposed to mitigate anthracycline-induced cardiotoxicity. Mechanistically, these agents effectively suppress doxorubicin-triggered, ZAK-mediated hyperactivation of the canonical MAPK cascades, thereby stalling the immediate execution of cardiomyocyte apoptosis and curtailing pathological remodeling (Dent, 2013; Wong et al., 2013). Similarly, in oncological settings, inhibition of ZAK by compounds such as PLX4720 has been shown to promote the apical extrusion of transformed epithelial cells, thereby facilitating their elimination from epithelial tissues (Maruyama et al., 2020). Furthermore, it was recently revealed that certain experimental inhibitors designed for the canonical Integrated Stress Response (ISR), such as the GCN2 inhibitor GCN2iB, exert their cytoprotective effects against UV radiation primarily through potent, direct inhibition of ZAK kinase rather than their intended target (Misra et al., 2026).

However, this high tissue-context dependency manifests as a profound “toxicity paradox” in clinical settings, creating a complex therapeutic dilemma. While such suppression of ZAK offers localized therapeutic benefits, it simultaneously abolishes intrinsic tumor-suppressive surveillance in the skin. Under physiological conditions, epidermal ZAKα serves as an indispensable sensor of UV-induced ribotoxic stress, actively driving JNK-mediated apoptosis and pyroptosis to eliminate damaged keratinocytes (Sinha et al., 2024). This paradox is most notably illustrated by targeted oncology therapies, such as the BRAF inhibitor vemurafenib. Neutralization of this localized ribotoxic stress pathway blocks the protective apoptotic response, underlying common clinical side effects. Even more severely, this blockade allows keratinocytes harboring UV-induced damage to evade cell death and undergo uncontrolled proliferation, ultimately driving the formation of cutaneous squamous cell carcinoma (cSCC) (Vin et al., 2014; Klövekorn et al., 2021; Mathea et al., 2016; Chapman et al., 2011; Bollag et al., 2012).

Given the current lack of highly selective ZAK inhibitors in the clinic, repurposing broad-spectrum kinase inhibitors requires carefully weighing on-target therapeutic efficacy against these severe “on-target, off-tissue” toxicities. Consequently, future drug development efforts must prioritize structure-guided approaches for isoform-selective targeting or localized delivery systems to minimize adverse effects while preserving the beneficial physiological functions of ZAK signaling.

### 5.2 Development of ZAK-Specific small molecules and allosteric strategies

To overcome the limitations associated with nonselective multi-kinase inhibitors, recent advances in structure-guided drug discovery have enabled the development of highly selective inhibitors targeting ZAK. In oncologic settings, particularly LUSC, the selective small-molecule inhibitor iZAK2 has demonstrated significant antitumor activity (Ye et al., 2023). Rather than acting merely as a general cytotoxin, its mechanism involves the precise decoupling of the DNA-PK/ZAK/AKT/mTOR signaling cascade. By disrupting this targeted axis, iZAK2 effectively impairs metabolic reprogramming, directly depriving tumor cells of the glycolysis-derived biosynthetic intermediates required to sustain aggressive proliferation and survival (Wang et al., 2024; Ye et al., 2023). Additional progress has been made with the design of covalent inhibitors that exploit structural features unique to the ZAK kinase domain. Specifically, compounds targeting the cysteine-22 (Cys22) residue within the ATP-binding pocket have shown nanomolar inhibitory potency in preclinical models. These molecules form irreversible covalent bonds with ZAK, resulting in durable suppression of downstream signaling pathways mediated by this kinase (Dittus et al., 2017).

Selective inhibition of ZAK is also being explored for non-oncologic diseases. A notable example includes 1,2,3-triazole benzenesulfonamide derivatives, such as Compound 3h, which display exceptional specificity for ZAK relative to other MAP3K family members. In spontaneously hypertensive animal models, oral administration of these compounds markedly reduces left ventricular hypertrophy and myocardial structural disorganization, demonstrating their therapeutic potential in cardiovascular disease (Chang et al., 2017; Jiang et al., 2019). Similarly, pharmacological agents such as Compound 6p have been shown to significantly decrease ECM accumulation in experimental models of ischemic and obstructive renal injury, suggesting possible applications in fibrotic kidney disorders (Shu et al., 2022). Beyond single-agent therapies, targeting ZAK may also enhance the efficacy of combination treatment strategies. In *TP53*-mutant triple-negative breast cancer, inhibition of ZAK has been reported to produce synthetic lethality when combined with Aurora Kinase B (AURKB) inhibition (Tang et al., 2019). In addition, in medulloblastoma models, ZAK inhibition prevents radiation-induced cell cycle arrest, acting as a potent radiosensitizer to further improve therapeutic outcomes (Markowitz et al., 2016). Importantly, it must be noted that while these highly selective ZAK antagonists demonstrate profound efficacy in experimental models, they remain strictly in the preclinical stage of development. Consequently, their dose-limiting toxicities, pharmacokinetic profiles, and ultimate clinical outcomes in human subjects have yet to be evaluated. Bridging this translational gap from animal models to human clinical trials represents a critical next step for ZAK pharmacology.

Due to the high conservation of the N-terminal kinase domains of ZAKα and ZAKβ, conventional ATP-competitive inhibitors inevitably cause pan-isoform inhibition. To overcome the aforementioned clinical dilemma to this non-discriminatory blockade, future drug discovery must transition from generic catalytic targeting to spatial and structural disruption. The recent cryo-EM elucidation of the ZAK-ribosome complex offers a precise structural blueprint for this paradigm shift. Instead of relying solely on targeting the highly conserved kinase pocket, exploring small-molecule inhibitors of protein-protein interactions (PPIs) at the ZAK-ribosome docking interface offers a conceptual alternative. Specifically, compounds designed to disrupt the interactions involving the receptor for activated C kinase 1 (RACK1)-interacting motifs (RIM and RIH) or the SAM domain dimerization interface unique to ZAKα could theoretically provide unprecedented selectivity (Vind et al., 2026). However, translating this concept into clinical candidates presents formidable biochemical challenges. PPI interfaces, including ribosome docking sites, are typically large, flat, and lack the deep, well-defined hydrophobic pockets characteristic of kinase active sites (Arkin and Wells, 2004). Furthermore, disrupting the highly dynamic and transient dimerization of the SAM domain requires potent allosteric modulators, which remain notoriously difficult to identify and optimize through traditional high-throughput screening (Huso et al., 2026; Vind et al., 2026). Transitioning from traditional kinome-wide screens toward these allosteric and interface-targeted strategies represents a challenging yet highly promising path to achieving true ‘on-target, on-tissue’ therapeutic precision.

### 5.3 Outstanding questions and mechanistic frontiers

Beyond pharmacological development, several major mechanistic gaps in ZAK biology remain to be addressed. For instance, while this stoichiometric heterodimerization provides an elegant biochemical explanation for tissue homeostasis, the dynamic regulation of its competitive equilibrium under fluctuating *in vivo* stress regimes remains obscure (Fu et al., 2016). In complex pathological microenvironments—such as the acute phase of myocardial ischemia or the heterogeneous tumor stroma—this stoichiometric balance is unlikely to remain static. A critical unanswered question is how parallel hyperactivated signaling networks within these milieus actively disrupt this equilibrium to drive a pathogenic phenotypic switch. We hypothesize that stress-induced post-translational modifications (PTMs), such as specific phosphorylation, ubiquitination, or SUMOylation events within the shared LZ motif, could dynamically alter heterodimeric binding affinity (Milde-Langosch, 2005; Nihalani et al., 2001). This could either dissolve protective heterodimers to unleash pathogenic ZAKα hyperactivation, or stabilize them to promote cell survival. Investigating these post-translational regulatory layers under shifting stress gradients represents a critical next step to move beyond descriptive, static models of isoform antagonism.

Another major uncharted territory for future research lies in the signaling bifurcation of ZAKα in tumor biology. Currently, the molecular mechanisms governing how ZAKα preferentially engages the non-canonical AKT/mTOR axis over its classical MAP2K/MAPK modules remain completely unresolved (Wang et al., 2024). We hypothesize that the DNA-PK complex does not merely act as a transient upstream kinase but functions as a dynamic molecular scaffold under specific microenvironmental pressures, reminiscent of how classical scaffold proteins (e.g., kinase suppressor of Ras (KSR) in the RAF/MEK/ERK pathway) orchestrate spatial signaling fidelity (Good et al., 2011; Kolch, 2005; Lavoie et al., 2018). As an illustration, localized oxidative stress or distinct metabolic shifts might alter the spatial conformation of this complex, physically sequestering ZAKα to prevent the homodimerization required for canonical RSR/MAPK activation, while simultaneously facilitating its proximity to AKT. Deciphering whether specific adaptor proteins or unique PTM patterns dictate this spatial bifurcation will be essential to fully understand metabolic plasticity and overcome chemoresistance in aggressive malignancies.

## 6 Discussion

The pathobiology of ZAK is characterized by profound functional plasticity rather than a simple “pathogenic versus protective” paradigm. Mechanistically, it transitions between three distinct operational modes: an autonomous disease driver when structurally mutated, a stress-response amplifier via sustained MAPK hyperactivation, and a contextual signaling node governed by microenvironmental inputs. Central to this versatility is the structural divergence of its splice variants. Recent insights have transformed our view of these isoforms from generic kinases to specialized cellular sensors at the crossroads of cellular stress: ZAKα relies on its specialized C-terminal domains to detect structural ribosomal collisions and utilizes its SAM domain to undergo collision-induced dimerization, orchestrating the RSR. Conversely, ZAKβ utilizes its SFBD to monitor cytoskeletal mechanical strain. This paradigm is powerfully underscored by human genetics, where the loss of ZAKβ mechanosensing drives congenital myopathies, while ZAKα SAM domain mutations cause developmental limb defects.

Translating this sophisticated kinase system into precision therapeutics remains a formidable challenge. Although existing broad-spectrum kinase inhibitors can suppress ZAK activity, their clinical use is inherently limited by severe “on-target, off-tissue” toxicities, highlighting the necessity for a paradigm shift in pharmacological targeting. Moving forward, a challenging yet conceptually critical Frontier lies in exploring structure-guided, isoform-selective interventions. Despite the significant biophysical hurdles of drugging flat PPI interfaces, developing allosteric molecules targeting the ZAK-ribosome docking interface or the SAM domain holds the theoretical potential to selectively decouple ZAKα from pathological stress cascades. Crucially, this approach neutralizes aberrant signaling while preserving both essential ZAKβ-mediated mechanotransduction and ZAKα′s intrinsic protective roles, such as epidermal tumor surveillance. Furthermore, resolving the dynamic regulation of ZAKα/ZAKβ heterodimerization under shifting *in vivo* stress gradients, as well as defining the structural basis for ZAKα′s spatial bifurcation toward the non-canonical AKT/mTOR metabolic axis, represent vital mechanistic imperatives. Ultimately, deciphering these regulatory layers and moving beyond broad-spectrum catalytic blockade will unlock the full therapeutic potential of targeting ZAK in complex human diseases.
